# Metabolomics reveals impaired maturation of HDL particles in adolescents with hyperinsulinaemic androgen excess

**DOI:** 10.1038/srep11496

**Published:** 2015-06-23

**Authors:** Sara Samino, Maria Vinaixa, Marta Díaz, Antoni Beltran, Miguel A. Rodríguez, Roger Mallol, Mercedes Heras, Anna Cabre, Lorena Garcia, Nuria Canela, Francis de Zegher, Xavier Correig, Lourdes Ibáñez, Oscar Yanes

**Affiliations:** 1 Spanish Biomedical Research Centre in Diabetes and Associated Metabolic Disorders (CIBERDEM), C/ Monforte de Lemos 3-5, 28029 Madrid, Spain; 2 Department of Electronic Engineering, Rovira i Virgili University, Avinguda Països Catalans 26, 43007 Tarragona, Spain; 3 Centre for Omic Sciences (COS), Rovira i Virgili University, Avinguda Universitat 3, 43204 Reus, Spain; 4 Research Unit on Lipids and Atherosclerosis, Sant Joan University Hospital, Universitat Rovira i Virgili, IISPV, Carrer Sant Llorenç 21, 43201 Reus, Spain; 5 Endocrinology Unit, Hospital Sant Joan de Déu, University of Barcelona, Passeig de Sant Joan de Déu 2, 08950 Esplugues, Barcelona, Spain; 6 Paediatric Endocrinology, University Hospital Gasthuisberg, UZ Herestraat 49, 3000 Leuven, University of Leuven, Belgium.

## Abstract

Hyperinsulinaemic androgen excess (HIAE) in prepubertal and pubertal girls usually precedes a broader pathological phenotype in adulthood that is associated with anovulatory infertility, metabolic syndrome and type 2 diabetes. The metabolic derangements that determine these long-term health risks remain to be clarified. Here we use NMR and MS-based metabolomics to show that serum levels of methionine sulfoxide in HIAE girls are an indicator of the degree of oxidation of methionine-148 residue in apolipoprotein-A1. Oxidation of apo-A1 in methionine-148, in turn, leads to an impaired maturation of high-density lipoproteins (HDL) that is reflected in a decline of large HDL particles. Notably, such metabolic alterations occur in the absence of impaired glucose tolerance, hyperglycemia and hypertriglyceridemia, and were partially restored after 18 months of treatment with a low-dose combination of pioglitazone, metformin and flutamide.

Hyperinsulinemic androgen excess (HIAE) is now recognized as the phenotypic core of a broader pathological entity traditionally known as polycystic ovary syndrome (PCOS)^1^,^2^,^3^,^4^, which affects 8–21% of women of reproductive age worldwide^5^,^6^,^7^. HIAE is a hallmark already present in both obese and non-obese adolescent girls^8^,^9^ and precedes a phenotype in adulthood characterized by anovulatory infertility, metabolic syndrome, type 2 diabetes (T2D)^10^ and possibly cardiovascular disease (CVD)^11^,^12^. HIAE girls, therefore, provide an opportunity for discovering metabolic derangements that can bring a better understanding of these long-term health risks in their early stages. In turn, these findings might prompt early therapeutic interventions in adolescent girls, thereby decreasing subsequent metabolic complications related to HIAE^4^.

In this context, mass spectrometry (MS) and nuclear magnetic resonance (NMR)-based metabolomics have the potential to discover metabolic alterations in clinical samples. Metabolomics enables the characterization of metabolites, the chemical entities that are transformed during metabolism and provide a functional readout of cellular biochemistry^13^. Global metabolite profiling studies are revealing new discoveries linking cellular pathways to biological mechanism, shaping our understanding of cell biology, physiology and medicine^14^,^15^,^16^.

Here we develop a comparative MS and NMR metabolomic analysis using serum samples of HIAE girls, and age-, weight- and ethnicity-matched healthy controls. Our goal is to identify as yet undefined metabolic alterations in HIAE that may help to understand the development of metabolic syndrome and T2D in adulthood.

Our results revealed an imbalance between pro-oxidant and anti-oxidant events in HIAE, which leads to oxidation of apolipoprotein A1 and impaired maturation of HDL particles. Notably, these metabolic derangements precede essential contributory factors to the metabolic syndrome and T2D, including impaired glucose tolerance, hyperglycemia or hypertriglyceridemia. We also demonstrate that a low-dose combination of pioglitazone, flutamide and metformin (PioFluMet) during 18 months restores the levels of oxidative markers to similar levels found in healthy girls.

## Results

### Endocrine and metabolic alterations associated with HIAE girls

Endocrine and metabolic parameters were compared between a group of 12 young, non-obese HIAE girls and 14 age-, weight- and ethnicity-matched healthy controls. Table 1 shows mean values and the standard error of the mean (SEM) for each variable. As expected by definition, endocrine alterations in adolescent HIAE girls relative to healthy controls included increased significant (p ≤ 0.05) serum levels of insulin, testosterone, dehydroepiandrosterone sulfate (DHEAS) and leptin. On the other hand, the fasting glucose to insulin ratio was decreased in HIAE girls. Levels of superoxide dismutase (SOD), a key antioxidant enzyme protecting the cells, were also decreased in HIAE girls. Triglycerides, LDL-cholesterol and HDL-cholesterol levels did not show differences between HIAE and healthy control girls.

Thus, hyperinsulinemia with normal fasting glucose levels in HIAE girls may reflect insulin resistance, as suggested by the increased ratio of glucose to insulin. However, we could observe no evidence of impaired glucose tolerance, hyperglycemia or hypertriglyceridemia at this early age.

### Alteration of the VLDL, LDL and HDL serum profile in HIAE girls

**Figure 1:**
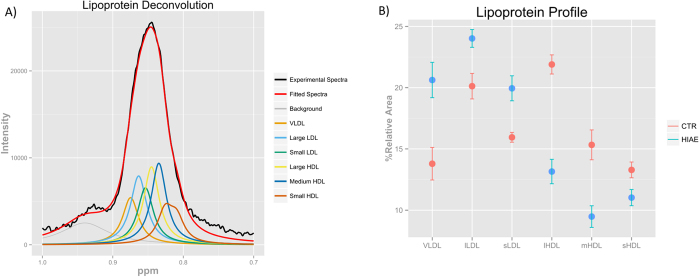
Lipoprotein profile measured by NMR spectroscopy. (**A**) Bipolar LED pulse sequence ^1^H NMR spectra of a HIAE serum showing the fitting of the methyl band using seven Lorentzian functions derived from our previously described methodology^18^. (**B**) The amount of lipoprotein particles is expressed as the percentage of particles for every lipoprotein subclass with regard to the total number of particles. Row-wise normalized areas showed as mean±sem. VLDL: very low-density lipoprotein, lLDL: large low-density lipoprotein, sLDL: small low-density lipoprotein, lHDL: large high-density lipoprotein, mHDL: medium high-density lipoprotein, sHDL: small high-density lipoprotein

Dyslipidemia is a risk factor linked to metabolic syndrome, T2D and CVD^17^. Here we studied the lipoprotein profile of HIAE girls beyond the standard measurement of cholesterol content of lipoproteins. By using an advanced lipoprotein analysis based on nuclear magnetic resonance (NMR) spectroscopy^18^, ^1^H-NMR allow to characterize the size and relative abundance of lipoprotein particles in serum. The use of NMR-derived lipoprotein subclasses improved cardiovascular risk stratification for subclinical atherosclerosis in comparison to conventional lipids^19^. Briefly, depending on the size of the lipoprotein particle, the methyl moieties of the lipids in lipoproteins resonate at slightly different frequencies, the smaller particles resonating at lower frequencies (Fig. 1a). Our NMR-derived lipoprotein subclasses were defined as VLDL, large LDL, small LDL, large HDL, medium HDL, and small HDL.

Relative levels of VLDL, small LDL and large LDL were significantly increased in HIAE relative to control girls. In contrast, the relative abundance of large, medium and small HDL subclasses was decreased in HIAE, with the greatest decline associated with large HDL (Fig. 1b). Supplementary Table 1 shows the differences in serum lipoproteins between HIAE girls and healthy controls. The characterization of lipoprotein subclasses by NMR revealed a dyslipidemic profile in HIAE girls, which is similar to the one observed in the metabolic syndrome and its associated pathologies; T2D and CVD^20^.

### Metabolomics reveals elevated levels of methionine sulfoxide and altered glutathione biosynthesis in HIAE girls

Our LC-qTOF MS and MS/MS-based untargeted metabolomic approach revealed lower levels of methionine in serum samples of HIAE girls, which were associated with greater levels of methionine sulfoxide (MetOx) in these patients, indicating increased oxidation of this amino acid (Supplementary Table 2). Furthermore, levels of γ-glutamyl dipeptides were increased in HIAE girls. γ-glutamyl dipeptides are involved in the γ-glutamyl cycle transporting amino acids into cells and synthesizing reduced glutathione (GSH), a key reducing agent that protects cells from oxidative damage. The NMR analysis, in addition, revealed lower levels of glycine in HIAE girls (Supplementary Table 3). Glycine can be added to the C-terminal of γ-glutamylcysteine (Glu-Cys) via the enzyme glutathione synthetase to produce GSH.

**Figure 2:**
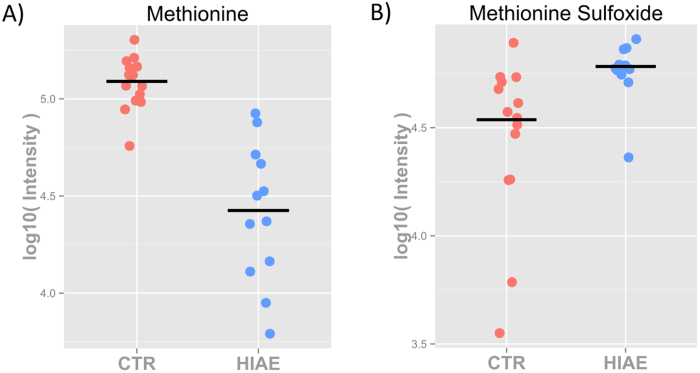
Targeted LC-QqQ MS analysis of methionine and methionine sulfoxide. The scatter plots show the abundance of methionine (**A**) and methionine sulfoxide (**B**) in controls and HIAE serum samples and trimmed mean (*P *< 0.05) (controls are depicted in red and HIAE in blue).

Next, all these changes in metabolites were validated and complemented with additional metabolites by targeted analyses of the serum samples using triple-quadrupole mass spectrometry (QqQ MS) with multiple reaction monitoring (MRM) (when chemical standards were commercially available) (Table 2). We confirmed that a drop in methionine levels (Fig. 2A) is accompanied by an increase in MetOx (Fig. 2B) in HIAE girls. To discard that an artifactual conversion of methionine to MetOx during sample preparation and analysis^21^,^22^ could be the cause of the increased levels of MetOx in HIAE girls, we spiked the same amount of free deuterium labelled methionine (S-methyl-d3) in HIAE and control serum samples, followed by LC-QqQ MS detection. Supplementary Fig. 1 shows no significant differences in the levels of methionine-d3 or the artifactually-produced methionine-d3 sulfoxide (MetOx-d3), nor in the MetOx-d3/Met-d3 ratio, between HIAE and control samples. In both scenarios the percentage of artifactual MetOx-d3 represents less than 2% of the total methionine-d3 signal. Therefore, the minimal artifactual oxidation of methionine in our method does not impact on the relative and biological differences observed between HIAE and the control group.

**Figure 3:**
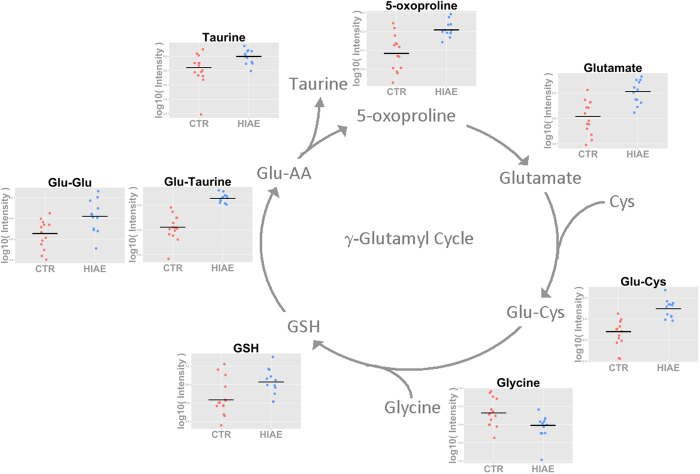
Identified and quantified metabolites involved in the γ-glutamyl cycle. The scatter plots show the abundance of individual metabolites in control and HIAE serum samples and trimmed mean (*P *< 0.05) (control in red and HIAE in blue). Glycine was measured by 1H-NMR and the other metabolites by LC-QqQ MS.

The biosynthesis of glutathione (by quantifying reduced glutathione), the redox status (by quantifying the ratio of reduced to oxidized glutathione) and intermediates of the γ-glutamyl cycle were also elevated in HIAE girls (Fig. 3). These data demonstrate a redox dysregulation in HIAE girls.

As a result of the redox dysregulation, methionine residues in proteins can be oxidized by reactive oxygen species to MetOx^23^. Oxidation of methionine residues in apolipoprotein-A1 (apo-A1) has been associated with impaired reverse cholesterol transport by HDL, and consequently, impaired maturation of HDL particles^24^. This could partly explain the lower percentage of HDL particles, and more specifically, of large HDL particles in girls with HIAE (Fig. 1B). Since apo-A1 is the major protein component of HDL and one of the most abundant proteins in human serum^25^, we hypothesized that increased levels of free MetOx in HIAE serum may reflect greater oxidation of methionine residues in apo-A1.

### Quantitative analysis of the MetOx-148/Met-148 ratio in apo-A1 by MALDI-TOF MS

To test the hypothesis that HIAE girls suffer from increased oxidation of methionine residues in apo-A1, we measured the ratio of MetOx/Met in apo-A1 using SDS-PAGE and MALDI-TOF MS (see the Methods section for details). In particular, we focused on a single methionine residue of apo-A1, Met-148, the oxidation of which has been associated with loss of lecithin cholesterol acyltransferase (LCAT) activity, a critical early step in reverse cholesterol transport^26^.

Apo-A1 sequence coverage was 61% on average and MetOx-148/Met-148 was calculated from the ratio of peak intensity of peptides m/z 1411.67 and m/z 1047.51 (sequence K.LSPLGEEMR.D). The ratio MetOx-148/Met-148 of apo-A1 was measured separately for each sample to minimize the spot-to-spot variability in MALDI MS. It is possible that the level of artifactual oxidation of Met-148 in this peptide could be greater than that of free methionine described above. However, based on Suppl. Fig. 1, we consider that the amount of artifactually-produced MetOx-148 from HIAE and control serum samples is similar, and consequently, it does not impact on the relative differences between groups.

**Figure 4:**
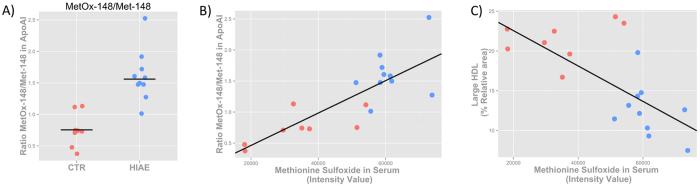
Oxidation of Apo-A1 and correlation of measurements. (**A**) Ratio of MetOx-148/Met-148 in apo-A1 (*P *< 0.05) measured by MALDI-TOF MS from the intensity of peptide K.LSPLGEEMR.D. (**B**) Statistically significant positive correlation (*P *= 6.95E-05 and r = 0.8) between free methionine sulfoxide in serum, as measured by LC-QqQ MS, and the oxidation state of apo-A1. (**C**) Statistically significant negative correlation (*P *= 1.13E-03 and r = −0.7) between free levels of methionine sulfoxide in serum and the percentage of large HDL particles, as measured by NMR. Controls are depicted in red and HIAE in blue.

The ratio MetOx-148/Met-148 in apo-A1 was significantly increased in HIAE girls compared with healthy controls (Fig. 4A). Furthermore, we found a positive and significant correlation (p = 6.95E-05 and r = 0.8) between the ratio MetOx-148/Met-148 in apo-A1 and free methionine sulfoxide in serum (Fig. 4B). Levels of methionine sulfoxide in serum, therefore, correlate with the degree of oxidation of the Met-148 residue in apo-A1, which may reflect turnover and proteolytic degradation of apo-A1 proteins. Finally, we found a negative correlation (p = 1.13E-03 and r = −0.7) between the number of large (i.e., mature) HDL particles and methionine sulfoxide in serum (Fig. 4C), which reinforces our hypothesis that levels of methionine sulfoxide in serum reflect HDL oxidation, and indirectly, impaired maturation of HDL particles.

### Metabolic changes after 18 months of PioFluMet polytherapy in HIAE girls

Ibáñez and colleagues demonstrated that a low-dose combination of pioglitazone, flutamide and metformin (PioFluMet) proved to be more beneficial than oral contraceptives in regulating endocrine-metabolic parameters, decreasing inflammation and visceral and hepatic fat, and in improving markers of cardiovascular health^27^.

Here, the PioFluMet polytherapy resulted in a reduction in insulin concentrations (Supplementary Fig. 2). We also measured if the novel metabolic markers described above in HIAE girls are subject to regulation by the PioFluMet polytherapy. Metabolites involved in the biosynthesis of GSH via the γ-glutamyl cycle, the size and relative abundance of lipoprotein particles, and the degree of oxidation of Apo-A1 through the MetOx-148/Met-148 ratio were monitored using NMR and MS in the serum of 6 patients after 18 months of PioFluMet treatment.

**Figure 5:**
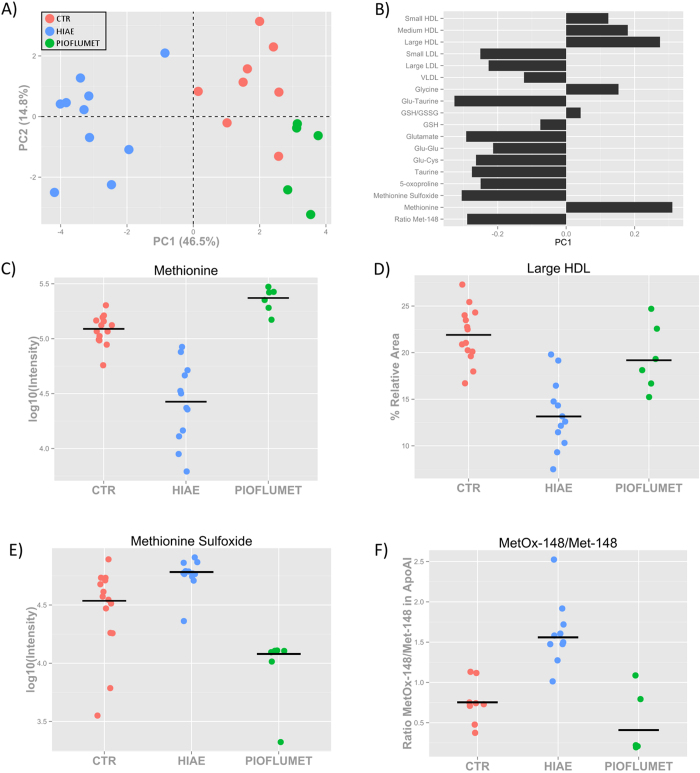
Metabolic changes after 18 months of PioFluMet polytherapy. (**A**) PC1/PC2 scatter scores plot and (**B**) PC1 loading bar plot of PCA showing all the metabolites measured in HIAE girls after the treatment. (**C**) Relative intensity of free methionine in serum as measured by LC-QqQ MS. (**D**) Percentage of large HDL particles in serum as measured by NMR. (**E**) Relative intensity of free methionine sulfoxide in serum as measured by LC-QqQ MS. (F) Ratio of MetOx-148/Met-148 in apo-A1 calculated from the intensity of the pepide K.LSPLGEEMR.D as measured by MALDI-TOF MS.

The abundance of individual markers was scaled to unit variance and projected using an unsupervised principal component analysis (PCA) (Fig. 5A). The scores plot reveals two clusters along PC1 (~47% of the variance) corresponding to HIAE girls on the one hand, and healthy controls and HIAE girls treated with PioFluMet on the other. This distribution indicates that the metabolic state of HIAE girls after 18 months of PioFluMet treatment more closely resembles the state of healthy girls, suggesting an overall improvement of the metabolic derangements in these girls. To interpret the pattern displayed in the scores plot, Fig. 5B shows a loading bar plot of the PCA using each individual metabolic marker. The relative abundance of large HDL particles and levels of free methionine in serum are the two largest contributing variables to positive values in PC1. After the treatment with PioFluMet, the levels of methionine and large HDL particles recovered to similar levels found in healthy girls (Fig. 5C,D). Likewise, the levels of methionine sulfoxide and the oxidation of Apo-A1 in the Met-148 residue decreased after the treatment, reaching similar or even lower levels than those seen in healthy girls (Fig. 5E,F).

## Discussion

A better understanding of hyperinsulinemic androgen excess (HIAE) in non-obese adolescents may help to identify early causes of pre-diabetic states^28^ and possibly cardiovascular events^29^ in adulthood. Ultimately, novel insights should sharpen the perspective of early HIAE prevention^4^.

Our study indicates an imbalance between pro-oxidant and anti-oxidant mechanisms in HIAE girls. The pro-oxidant events include increased oxidation of methionine residues in apo-A1 and accumulation of the oxidative marker methionine sulfoxide in HIAE serum. Interestingly, these patients appear to have the capacity to activate a compensatory anti-oxidant mechanism that probably aims at regulating their redox status by synthesizing glutathione (GSH) through the γ-glutamyl cycle^30^. Redox imbalances have been also described in adult women with a PCOS phenotype^31^,^32^,^33^, atherosclerotic diseases^34^, diabetes^35^,^36^ and metabolic syndrome^37^. However, the underlying causes of these associations remain unclear.

High-density lipoprotein (HDL) has protective activities against atherosclerosis, including its role in reverse cholesterol transport^38^,^39^. Its antioxidant and anti-inflammatory functions^40^ are generally associated with lower risk of cardiovascular disease^41^, metabolic syndrome and T2D^42^,^43^,^44^. The functional status of HDL is closely linked to its primary protein component, apo-A1. Oxidation of methionine residues in apo-A1 has been shown to impair reverse cholesterol transport by HDL^24^. Specifically, the oxidation of Met-148 in apo-A1 impairs apo-A1’s ability to activate LCAT^24^. LCAT is the enzyme responsible for converting free cholesterol into cholesteryl ester, which is then sequestered into the core of a lipoprotein particle, transforming newly synthesized HDL into spherical HDL. In our study, the increased oxidation of Met-148 residues in apo-A1 and the lower percentage of large (i.e., mature) HDL particles observed in HIAE girls, suggest impaired HDL function.

Similarly, recent experimental studies point out that the concentration of HDL particles, rather than the cholesterol carried by these particles, might be the appropriate parameter for assessing the function of HDL^45^. The HDL-cholesterol levels in HIAE girls within normal limits and the lower concentration of HDL particles in these girls, as measured by routine enzymatic colorimetric methods and NMR, respectively, might suggest higher levels of cholesterol-overloaded HDL particles^46^, with potentially less capacity for cholesterol efflux. Further work is needed to explore the link between oxidation of methionine residues in apo-A1, cholesterol-overloaded HDL particles and impaired HDL functions.

**Figure 6:**
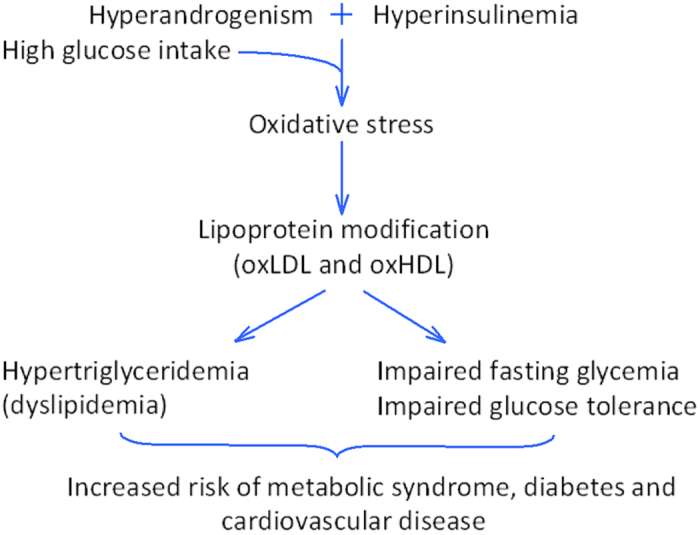
Proposed mechanism to explain the long-term health risks of hyperinsulinaemic androgen excess. Schematic representation of the underlying pathway by which HIAE is associated with long-term health risks, namely metabolic syndrome, diabetes and cardiovascular disease.

Recently, Lee *et al* demonstrated that low sugar consumption is associated with increasing HDL levels in females during adolescence^47^. In addition, excessive sugar intake in combination with hyperandrogenism causes oxidative stress^48^,^49^,^50^. In this context, we postulate that the current period of caloric abundance and chronically positive energy balance for most adolescents^51^, induces oxidation of lipoprotein particles and impaired lipoprotein function. Our data and those of others^52^,^53^, indicate that alterations in lipoprotein metabolism precede impaired fasting glycemia, impaired glucose tolerance and hypertriglyceridemia (Fig. 6). This suggests that the triad of methionine sulfoxide in serum, size of HDL particles and the ratio of methionine oxidation in apo-A1 may potentially become a novel biomarker of pre-diabetes and metabolic syndrome. Further work is needed to study this combination of biomarkers in the general population.

Finally, early treatment with a low-dose combination of PioFluMet proves again its efficacy in reverting androgen excess and hyperinsulinemia^54^, while improving the lipoprotein profile and levels of oxidative stress markers in HIAE girls. These observations extend further the benefits of therapies leading to a more physiological condition in HIAE girls, questioning the rationale for applying symptom-directed therapies that might potentially impact on later co-morbidities^55^.

## Methods

The methods were carried out in accordance with the approved guidelines of ISRCTN, which is a primary clinical trial registry recognised by WHO and ICMJE. The study was registered in Feb 2010 as ISRCTN45546616 (http://www.isrctn.com/ISRCTN45546616).

### Study population

The study population consisted of 12 non-obese adolescents (age, 16.3 ± 0.4 yr; BMI, 22.8 ± 0.5 Kg/m^2^) diagnosed with HIAE and 14 age-, weight- and ethnicity-matched healthy controls. HIAE girls were recruited at the Endocrinology Unit of the Sant Joan de Déu Children’s Hospital, Barcelona (Spain), among those randomized into a clinical study comparing the effects of a low-dose combination of Pioglitazone (7.5 mg/d) + Flutamide (62.5 mg/d) + Metformin (850 mg/d) (PioFluMet) with those of a frequently prescribed oral contraceptive [cyproteroneacetate 2 mg + 35 mcg ethynilestradiol for 21 of 28 d, and placebo for 7 of 28 d]^54^. The girls were chosen among those having enough serum sample left to allow for the required assessments. Controls were recruited among age-matched student mates with no clinical signs of androgen excess and regular menstrual cycles.

Inclusion criteria were: 1) hyperinsulinemia, defined as fasting-insulinemia above 150U/ml and/or hyperinsulinemia on a standard 2-h oral glucose tolerance test, defined as peak insulin levels >150 U/mL and/or mean serum insulin >84 μU/mL; and 2) the presence of both clinical and biochemical androgen excess, as defined by the following: hirsutism score above 8 (Ferriman-Gallwey), amenorrhea (no menses for 3 months) or oligomenorrhea (menstrual cycles longer than 45 d); and increased levels of circulating androstenedione and/or testosterone in the follicular phase of the cycle (d 3–7) or after 2 months of amenorrhea^54^.

Exclusion criteria were: evidence of anemia, thyroid dysfunction, bleeding disorder, Cushing syndrome, or hyperprolactinemia; glucose intolerance; diabetes mellitus; late-onset adrenal hyperplasia; abnormal electrolytes; abnormal screening of liver or kidney functions; use of medication affecting gonadal or adrenal function, or carbohydrate or lipid metabolism. Pregnancy risk was a particular exclusion criterion that was not only taken into account at study start, but was also maintained throughout the study in the PioFluMet subgroup.

### Ethics

This clinical study was registered as ISRCTN12871246 and conducted in Sant Joan de Déu University Hospital (Barcelona, Spain), after approval by the Institutional Review Board of Sant Joan de Déu University Hospital, and after written informed consent by the parents and assent by each participating girl.

### Global metabolomics profiling

Untargeted metabolomic analyses on serum samples of HIAE and control girls were performed using two analytical platforms: ^1^H-NMR and LC-ESI-QTOF. Each serum sample was split into two aliquots and run in parallel using the two analytical platforms. For the NMR measurement 250 μL of serum were mixed with 250 μL of phosphate buffer (0.75 mM Na_2_HPO_4_ adjusted at pH 7.4, and 20% D_2_O to provide the field frequency lock). The final solution was transferred to a 5 mm NMR tube and kept refrigerated at 4 °C in the autosampler until the analysis. ^1^H-NMR spectra were recorded at 310 K on a Bruker Avance III 600 spectrometer operating at a proton frequency of 600.20 MHz using a 5 mm CPTCI triple resonance (^1^H, ^13^C, ^31^P). Two different ^1^H-NMR pulse experiments were performed for each sample: 1) Nuclear Overhauser Effect Spectroscopy (NOESY)-presaturation sequence to suppress the residual water peak; and 2) Carr-Purcell-Meiboom-Gill sequence (CPMG, spin-spin T2 relaxation filter) with a total time filter of 410 ms to attenuate the signals of serum macro-molecules to a residual level.

The second aliquot was used for LC-MS analysis. 30 μL of serum sample was mixed with 120 μL of cold ACN/H_2_O (1:1) with 1% meta-phosphoric acid (MPA) and 0.1% formic acid (previously filtered), a simple and rapid extraction method particularly indicated for minimizing oxidation of redox species^56^,^57^. Samples were vortexed vigorously for 30 seconds and stored at –20 °C for 2 hours to enable protein precipitation. Subsequently, samples were centrifuged 15 minutes at 4 °C and 22600 RCF and the supernatant was transferred to a LC-MS vial. Samples were injected in a UHPLC system (1290 Agilent) coupled to a quadrupole time of flight (QTOF) mass spectrometer (6550 Agilent Technologies) operated in positive electrospray ionization (ESI+) mode. Metabolites were separated using either C18-RP (ACQUITY UPLC HSS T3 1.8 μm, Waters) or HILIC (ACQUITY UPLC BEH 1.7 μm, Waters) chromatography at a flow rate of 0.4 mL/min. The solvent system in C18-RP was A = 0.1% formic in water, and B = 0.1% formic in acetonitrile. The linear gradient elution started at 100% A (time 0–3 min) and finished at 100% B (20–21 min). The solvent system in HILIC was A = 50mM NH_4_OAc in water, and B= ACN. The linear gradient elution started at 95% B (time 0–2 min) and finished at 55% B (6 min). The injection volume was 2 μL. ESI conditions were gas temperature, 225 °C; drying gas, 13 L min^–1^; nebulizer, 20 psig; fragmentor, 125 V; and skimmer, 65 V. The instrument was set to acquire over the *m*/*z* range 80–1200 with an acquisition rate of 4 spectra/s. Quality control samples (QC) consisting of pooled serum samples of all patients were used. QC samples were injected before the first study sample and then periodically after five-study samples. Furthermore, real samples were randomized to reduce systematic error associated with instrumental drift. MS/MS was performed in targeted mode, and the instrument was set to acquire over the *m*/*z* range 50–1000, with a default iso width (the width half-maximum of the quadrupole mass bandpass used during MS/MS precursor isolation) of 4 *m*/*z*. The collision energy was fixed at 20 V. With the exception of glutamyl taurine and glutamyl glycine, all metabolites were identified conforming to Level 1 as specified by the Metabolomics Standards Initiative^58^, that is, by comparison with accurate mass, retention time and MS/MS data of chemical standards analysed in our laboratory with the same analytical platform and method. Glutamyl taurine and glutamyl glycine conform Level 2 (i.e., putatively annotated compound) since we did not find the chemical standards. Still, their experimental MS/MS spectra coincide with the expected fragmentation pattern of these dipeptides. Metabolomic data has been deposited in the MetaboLights database (accession code: MTBLS103).

### Targeted metabolomics

Relevant metabolites were measured again using an UHPLC system (1290 Agilent) coupled to a triple quadrupole (QqQ) MS (6490 Agilent Technologies) operated in multiple reaction monitoring (MRM) and positive electrospray ionization (ESI+) mode. MRM transitions were: methionine (150→ 56, 61), methionine sulfoxide (166→ 56, 74), taurine (126→ 41, 85), glutamate (148→56, 84), cysteine-glycine (179→ 59, 116), glutathione (308→ 76, 162), glutathione disulfide (613→ 355, 484), glutamate-glutamate (277→ 84, 130), glutamate-cysteine (251→ 84, 122), 5-oxoproline (130→ 56, 84).

### Spiking experiments with stable isotope labelled methionine

Free deuterium labeled methionine (S-methyl-d3) was spiked into HIAE and control serum samples at a final concentration 0,5 mM. Following metabolite extraction with meta-phosphoric acid (see details above), methionine-d3 and MetOx-d3 were measured using a LC-QqQ MS (6490 Agilent Technologies) in ESI+ and MRM mode. MRM transitions were: methionine-d3 (153→ 56, 64, 107), methionine-d3 sulfoxide (169→ 74, 56, 78).

### Characterization of lipoprotein classes

^1^H NMR spectra were recorded at 310 K on a Bruker Avance III 600 spectrometer operating at a proton frequency of 600.20 MHz (14.1 T). We used the double stimulated echo (DSTE) pulse program with bipolar gradient pulses of 1 ms and a gradient pulse strength of 95% of the maximum strength of 53.5 Gauss cm^−1^ in order to completely attenuate signals from low molecular weight metabolites. The relaxation delay was 2 s, the free induction decays (FIDs) were collected into 64 K complex data points and 32 scans were acquired on each sample. The methyl signal was line-shape fitted using eight 1D Lorentzian functions using a modification of a previously reported protocol^18^. According to the NMR-derived lipoprotein sizes previously described, functions 2 to 8 were associated with 1 VLDL, 2 LDL, and 4 HDL lipoprotein subclasses, respectively. For simplification, functions 7 to 8 were grouped to obtain the small HDL subclass.

### Data analysis and statistical methods

The acquired CPMG NMR spectra were phased, baseline corrected and referenced to the chemical shift of the α-glucose anomeric proton doublet at 5.23 ppm. Pure compound references in Bioref AMIX (Bruker), HMDB and Chenomx databases were used for metabolite identification. After baseline correction, intensities of each ^1^H-NMR regions identified in the CPMG 1D-NMR spectra were integrated for each sample entering the study using the AMIX 3.8 software package (Bruker, GmBH).

LC-MS (RP-C18 and HILIC ESI+ mode) data were processed using the XCMS software^59^ (version 1.38.0) to detect and align features. A feature is defined as a molecular entity with a unique m/z and a specific retention time. XCMS analysis of these data provided a matrix containing the retention time, m/z value, and integrated peak area of greater than 38.000 features after the analytical variability had been corrected^60^. Only the integrated areas of those metabolite features above 5,000 spectral counts in at least one of the two groups were considered for quantification. The tab-separated text files containing LC-MS data were imported into Rstudio (version 3.0.2) where QC samples were used to filter analytical variation as previously described^60^. Univariate statistical analysis was performed using robust statistics (Yuen-Welch’s t-test). Differentially regulated metabolites (fold > 1.5) that passed our statistical criteria (p-value < 0.01) were characterized by LC-qTOF MS/MS and identified using the Metlin database and pure standards purchased on our lab. Regression models and multivariate data analysis were performed using Rstudio software version 3.0.2.

### SDS-PAGE and trypsin digestion of apolipoprotein A1

Protein precipitation was carried out adding 10% of pure trichloroacetic acid (TCA) to 10 μL of serum. Samples were vortexed and incubated on ice for 1 hour. Samples were then centrifuged at 4 °C and 14.000 rpm for 15 minutes and the supernatants were discarded. 800 μL of cold acetone (−20 °C) were added to the pellet and proteins were suspended and incubated overnight (−20 °C). Samples were centrifuged and the supernatant discarded again. This step was repeated and the pellet was air-dried. Pellet was resuspended with 100 μL of urea (7M), thiourea (2M) and CHAPS (4%) buffer. 10 μL of this solution were added to 30 μL of Laemmli Buffer (4x). 40 μL of such solution was applied to a home-made 12% acrylamide/bis-acrylamide SDS-PAGE gel.

Proteins were Coomassie stained. The band of interest corresponding to Apo-A1 (MW: 28,1 kDa) was manually excised from 1D SDS-PAGE gels, destained and washed with 25 mM ammonium bicarbonate (AmBic) for 15 min followed by a wash with acetonitrile for 15 min. These washes were twice repeated and samples were finally dehydrated with 100% acetonitrile and dried in a Speed-Vack concentrator. Apo-A1 was cysteine carbamidomethylated by placing the dried gel at 56 °C for 1 h in a reducing solution containing 10 mM DTT and 50 mM AmBic. Alkylation of the cysteines was achieved by incubation of the gel for 30 min in the dark with 55 mM iodoacetamide in 25 mM AmBic buffer. Gel pieces were alternately washed with 25 mM AmBic and 25 mM AmBic with acetonitrile, and finally dehydrated with 100% acetonitrile and dried under vacuum. Gel pieces were incubated with 12.5 ng/μl sequencing grade trypsin (Roche Molecular Biochemicals) in 25 mM AmBic overnight at 37 °C. After digestion, the supernatants were separated. Peptides were extracted from the gel pieces into 50% ACN, 0.1% trifluoroacetic acid. For each extraction, samples were incubated for 10 min in an orbital shaker. All extracts were pooled and the volume reduced using a vacuum concentrator. In order to obtain a suitable sample for mass spectrometry analysis the pellet was resuspended in 25 μL of 0.1% TFA/water, desalted and concentrated using C18 ZipTips (Millipore). Tryptic peptides were sequentially eluted with 5 μL of 70% acetonitrile with 0.1% TFA in water.

### MALDI-TOF MS analysis of Apo-A1

Samples were spotted on the MALDI plate following the dried-droplet method. Briefly, 1 μl of the reconstituted in-gel digest sample was spotted on a BigAnchorChip target plate (Bruker Daltonics), followed by 1 μl of matrix (10 mg/ml α-cyano-4-hydroxycinnamic acid matrix (Bruker Daltonics) in 50% ACN, 0.1% TFA). Sample and matrix mixture was dried at room temperature. Mass spectra were obtained on an UltrafleXtreme (Bruker Daltonics, Bremen, Germany) matrix-assisted laser desorption ionization–tandem time of flight (MALDI-TOF/TOF) mass spectrometer. Mass spectra were recorded in positive ionization reflectron mode in the mass range of 700–3500 Da. Operating conditions were as follows: ion source 1 = 25.00 kV, ion source 2 = 24.40 kV, lens voltage = 8.50 kV, reflector voltage = 26.45 kV, optimized pulsed ion extraction time = 130 ns, matrix suppression = 500 Da. 1500 single-shot spectra were accumulated by recording 50-shot spectra at 10 random positions using fixed laser attenuation. Mass spectra were externally calibrated using a standard peptide mixture (Bruker); calibration was considered good when a value below 1 ppm was obtained.

### Peptide mass fingerprinting of Apo-A1 and quantization of the ratio MetOx-148/Met-148

ProteinScape software (Bruker) supported by the Mascot search engine (Matrix Science) was used with the following parameters: SWISS-PROT non-redundant database filtered by *Homo sapiens* taxonomy, two missed cleavage permission, 50-ppm measurement tolerance.

Carbamidomethylation of cysteines was set as a fixed modification and methionine oxidation was set as a variable modification. Positive identifications were accepted with a Mascot score higher than that corresponding to a *P* value of 0.05. The quantification of the ratio MetOx-148/Met-148 was performed using the intensity of the peptide K.LSPLGEEMS.D (Flex Analysis, Bruker).

## Additional Information

**How to cite this article**: Samino, S. *et al* Metabolomics reveals impaired maturation of HDL particles in adolescents with hyperinsulinaemic androgen excess. *Sci. Rep* **5**, 11496; doi: 10.1038/srep11496 (2015).

## Supplementary Material

Supplementary Information

## Figures and Tables

**Table 1:** Anthropometric and biochemical variables in patients and age- and BMI-matched controls.

	CTR	HIAE	p-value
Age (yr)	17.2 ± 0.4	16.3 ± 0.4	0.15
BW SDS	0.3 ± 0.1	-0.2 ± 0.4	0.58
Wt (kg)	58.8 ± 1.8	58.2 ± 1.2	0.92
Ht (cm)	163.9 ± 1.3	160.0 ± 1.5	0.06
BMI (Kg/m^2^)	21.8 ± 0.6	22.8 ± 0.5	0.23
BMI SDS	0.2 ± 0.2	0.5 ± 0.2	0.16
WBC (cell/mm^3^)	7.3 ± 0.3	7.8 ± 0.5	0.26
Neutrophils (x1000/mm^3^)	4.1 ± 0.3	4.4 ± 0.5	0.63
Lymphocytes (x1000/mm^3^)	2.2 ± 0.1	2.5 ± 0.22	0.41
N/L (ratio)	1.9 ± 0.2	2.0 ± 0.4	0.51
AST (μL/L)	16.6 ± 0.7	16.8 ± 1.6	0.59
ALT (μL/L)	13.6 ± 1.0	13.2 ± 1.1	0.83
Glucose (mg/dL)	89.1 ± 1.5	85.4 ± 2.0	0.14
oGTT MSG (mmol/L/h)	n.d.	6.7 ± 0.3	–
oGTT MSI (μU/L/h)	**n.d.**	45.9 ± 5.4	–
Insulin (μU/mL)	3.5 ± 0.6	10.3 ± 1.6	**0.01**
G/I ratio	32.8 ± 3.6	11.2 ± 1.9	**0.0004**
Total Cholesterol	143.9 ± 5.9	145.9 ± 6.8	0.75
HDL-cholesterol	52.6 ± 2.3	51.9 ± 3.3	0.77
LDL-cholesterol	80.5 ± 5.4	78.7 ± 4.5	0.88
Triglycerides	53.4 ± 3.6	76.8 ± 16.5	0.57
Testosterone (ng/dL)	32 ± 2.4	64.2 ± 10.2	**0.05**
DHEAS (μg/dL)	222.1 ± 27.8	280.8 ± 31.5	**0.03**
Leptin (ng/mL)	13.9 ± 2.3	20.9 ± 2.7	**0.05**
usCRP (mg/L)	0.7 ± 0.2	1.1 ± 0.2	0.14
SOD (U/mL)	6.1 ± 0.3	5.4 ± 0.2	**0.03**

Data are represented as mean ± (SEM). P-values are calculated from a robust Yuen-Welch’s t-test. BW SDS: Birth weight standard deviation score, Wt: weight, Ht: height, BMI: body mass index, BMI SDS: body mass index standard deviation score, WBC: white blood cells, N/L: Neutrophils/Lymphocytes, AST: aspartate transaminase, ALT: alanine aminotransferase, oGTT: oral glucose tolerance test, MSG: mean serum glucose, MSI: mean serum insulin, G/I ratio: Glucose/insulin ratio, HDL-cholesterol: high density lipoprotein – cholesterol, LDL-cholesterol: low density lipoprotein – cholesterol, DHEAS: dehydroepiandrosterone sulfate, usCRP: ultrasensitive-C-reactive protein, SOD: superoxid dismutase, n.d.: not determined

**Table 2:** Targeted metabolomics.

	% variation	p-value
Methionine	>−100	0.00005
Methionine Sulfoxide	40	0.0018
5-oxoproline	39	0.005
Taurine	28	0.015
Glu-Cys	37	0.0002
Glu-Glu	35	0.027
Glutamate	33	0.004
GSH	34	0.036
GSH/GSSG	29	0.023
Glu-Taurine*	>100	0.0000003
Glu-Gly*	71	0.00015

Percentage of variation and p-values (Yuen-Welch’s t-test) of metabolites. Negative and positive values indicate lower and higher levels, respectively, in girls with HIAE relative to healthy controls. Glu: glutamate, Cys: cysteine, GSH: glutathione, Gly: glycine, GSH/GSSG: glutathione/glutathione disulfide. *Glu-taurine and *Glu-Gly could not be quantified by LC-QqQ in MRM mode due to the lack of pure standards and reported values are from LC-qTOF MS.
