# Supplementary material for: Metabolomics reveals impaired maturation of HDL particles in adolescents with hyperinsulinaemic androgen excess
Source: Sci Rep. 2015 Jun 23;5:11496. doi: 10.1038/srep11496 (PMC4477239; doi:10.1038/srep11496)
Supplement: Supplementary Information [file srep11496-s1.doc]

**Metabolomics reveals impaired maturation of HDL particles in adolescents with hyperinsulinaemic androgen excess**

Sara Samino^1,3^, Maria Vinaixa^1,2,3^, Marta Díaz^1,5^, Antoni Beltran^1,3^, Miguel A. Rodríguez^1,3^, Roger Mallol^1,2^, Mercedes Heras^1,4^, Anna Cabre^1,4^, Lorena Garcia^3^, Nuria Canela^3^, Francis de Zegher^6^, Xavier Correig^1,2^, Lourdes Ibáñez^1,5*^, Oscar Yanes^1,2,3*^

1. Spanish Biomedical Research Centre in Diabetes and Associated Metabolic Disorders (CIBERDEM), C/ Monforte de Lemos 3-5, 28029 Madrid, Spain; 2. Department of Electronic Engineering, Rovira i Virgili University, Avinguda Països Catalans 26, 43007 Tarragona, Spain; 3. Centre for Omic Sciences (COS), Rovira i Virgili University, Avinguda Universitat 3, 43204 Reus, Spain; 4. Research Unit on Lipids and Atherosclerosis, Sant Joan University Hospital, Universitat Rovira i Virgili, IISPV, Carrer Sant Llorenç 21, 43201 Reus, Spain; 5. Endocrinology Unit, Hospital Sant Joan de Déu, University of Barcelona, Passeig de Sant Joan de Déu 2, 08950 Esplugues, Barcelona, Spain. 6. Paediatric Endocrinology, University Hospital Gasthuisberg, UZ Herestraat 49, 3000 Leuven, University of Leuven.

*To whom correspondence should be addressed.

| Oscar Yanes, PhD. Centre for Omic Sciences  Rovira i Virgili University Avinguda Universitat, 3.  43204 Reus (Spain) phone: [+34 977776617](tel:%2B34 977776617) email: [oscar.yanes@urv.cat](mailto:oscar.yanes@urv.cat) | Lourdes Ibañez, PhD-MD.  Hospital Sant Joan de Déu  University of Barcelona  Passeig de Sant Joan de Déu, 2.  08950 Esplugues, Barcelona (Spain).  Phone: +34 932804000; 4424  Email: [Libanez@hsjdbcn.org](mailto:Libanez@hsjdbcn.org) |
| --- | --- |

##

**
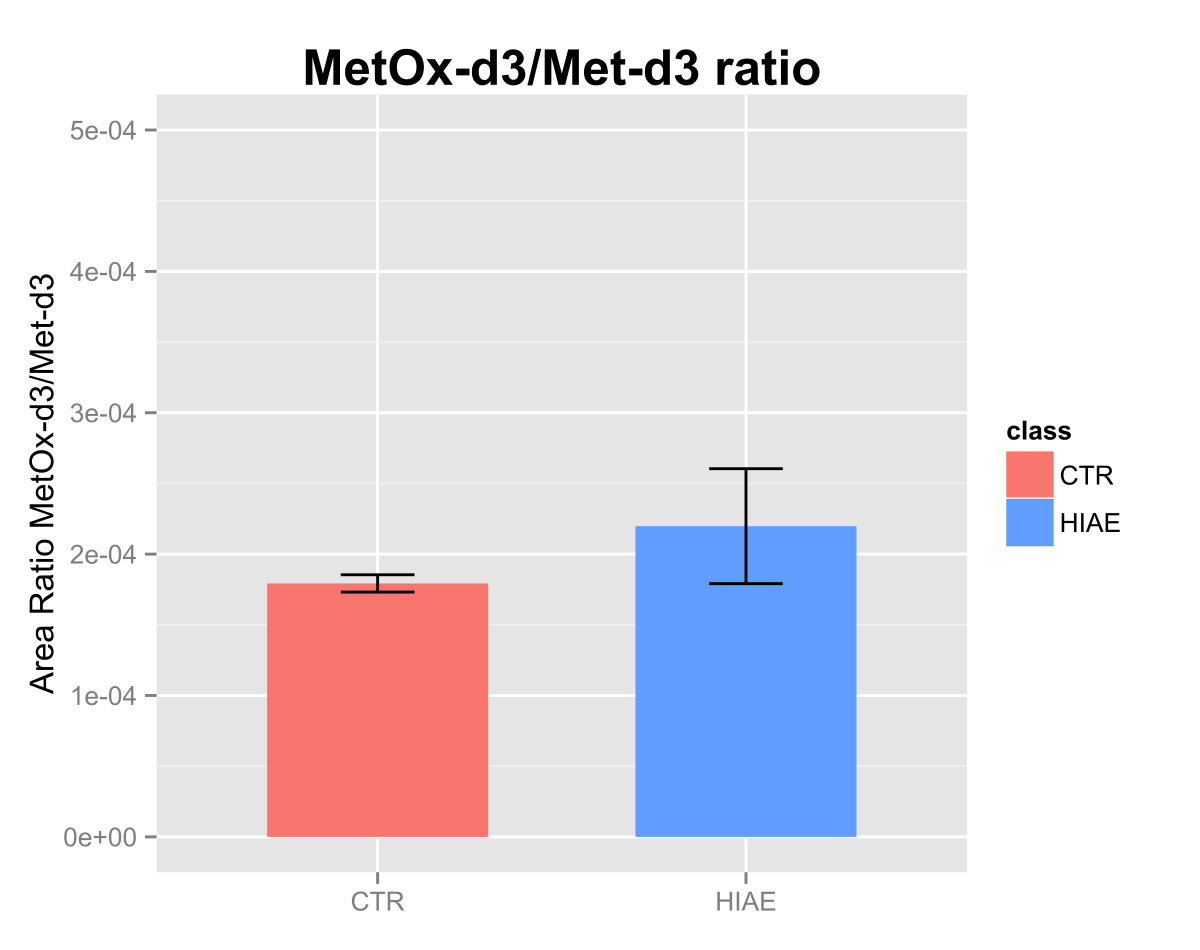
**

**Supplementary Figure 1. Ratio of free methionine-d3 sulfoxide/methionine-d3 in HIAE and control serum samples**. The ratio MetOx-d3/Met-d3 is calculated by integrating the area of the most intense MRM transition on a LC-QqQ MS for each metabolite. Data are represented as the mean ratio ± standard error of the mean. The p-value is calculated from a robust Yuen-Welch’s t-test (p-value=0.36).


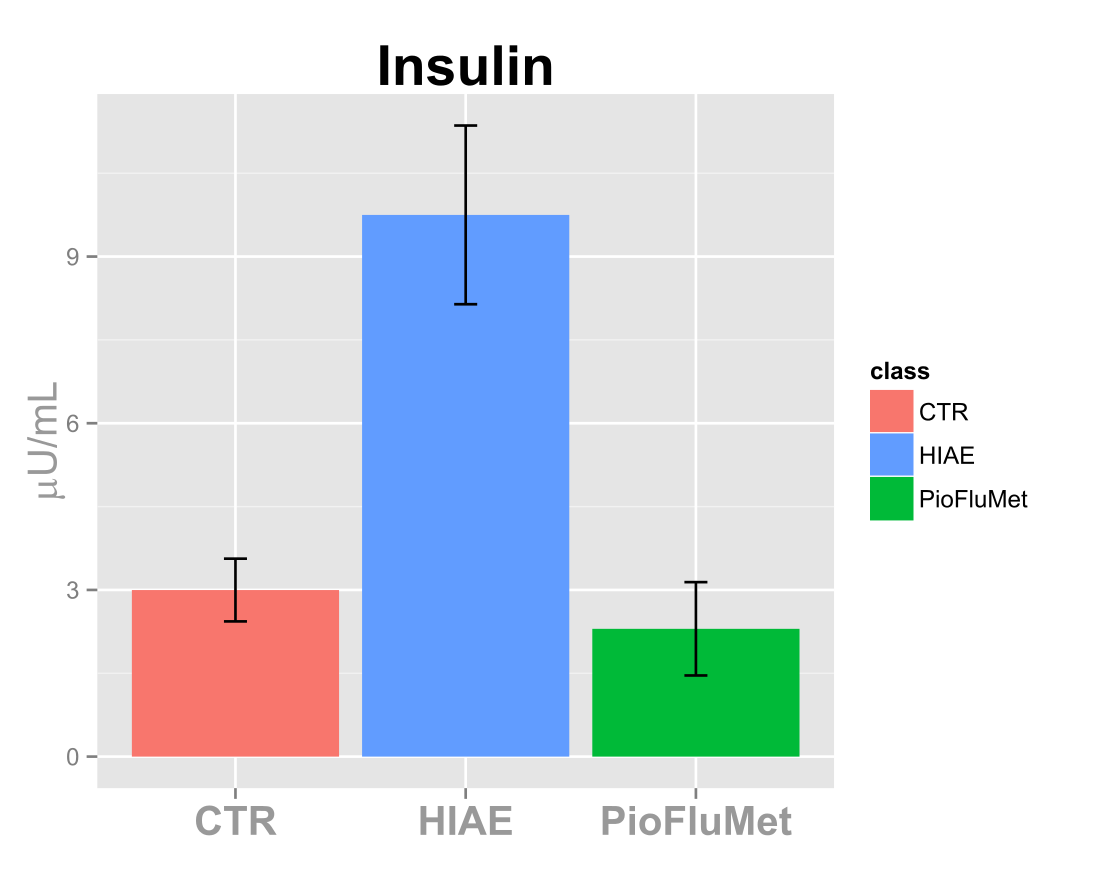


**Supplementary Figure 2. Serum insulin levels.** Data are represented as mean ± standard error of the mean. The p-values are calculated from a robust Yuen-Welch’s t-test and show statistically significant differences between CTR vs. HIAE (p<0.01) and HIAE vs. PioFluMet (p<0.05)

**Supplementary Table 1.** **NMR lipoprotein profile in serum samples*.*** Percentage of variation of each lipoprotein subclass in HIAE with respect to control girls, and p-values (Yuen-Welch’s t-test).

|  | % variation | p-value |
| --- | --- | --- |
| VLDL | 50 | 0.012 |
| Large LDL | 20 | 0.0264 |
| Small LDL | 31 | 0.0245 |
| Large HDL | -63 | 0.00001 |
| Medium HDL | -52 | 0.0058 |
| Small HDL | -20 | 0.02 |

**Supplementary Table 2.** **Untargeted LC-MS metabolomic experiment.** Metabolites identified by RP-C18 and HILIC that showed a variation greater than 50% between control and HIAE groups with a p-value < 0.01 (Yuen-Welch’s t-test).

|  | **% variation** | **p-value** | **UPLC Column** |
| --- | --- | --- | --- |
| Taurine | 52 | 0.0052 | HILIC |
| Glutamate | >100 | 0.004 | HILIC |
| Methionine sulfoxide | >100 | 0.00002 | HILIC |
| Choline | 51 | 0.0019 | HILIC |
| Methionine | >-100 | 0.0001 | HILIC |
| 5-oxoproline | 76 | 0.0028 | HILIC |
| Glutamine | -69 | 0.00015 | RP-C18 |
| Glu-Gly | 71 | 0.00015 | RP-C18 |
| Glu-Glu | 56 | 0.0031 | RP-C18 |
| Val-Glu | >100 | 0.00079 | RP-C18 |
| Glu-Taurine | >100 | 0.0000003 | RP-C18 |
| PC (16:1) | >100 | 0.000037 | HILIC & RP-C18 |
| PC (10:2) | >100 | 0.0005 | RP-C18 |

**Supplementary Table 3.** **NMR metabolic profile**. P-values were obtained from a Yuen-Welch’s t-test.

|  | **% variation** | **p-value** |
| --- | --- | --- |
| Lactate | 25 | 0.0092 |
| Acetoacetate | -20 | 0.1493 |
| Acetates | -10 | 0.2945 |
| Alanine | -3 | 0.6404 |
| Valine | 11 | 0.0792 |
| Isoleucine+Valine | -1 | 0.9184 |
| Leucine+Isoleucine | -23 | 0.1711 |
| Glucose | -9 | 0.0656 |
| Tyrosine | -19 | 0.1009 |
| Hystidine | -19 | 0.1951 |
| Glutamine | -56 | 0.0004 |
| Free Glycerol | -64 | 0.043 |
| Lysine | -47 | 0.0530 |
| Glutamate | 16 | 0.0717 |
| Creatine | 3 | 0.4382 |
| Citrate | -239 | 0.0187 |
| Glycine | -20 | 0.031 |
